# Highly Sensitive Electrochemical Detection of Paraquat in Environmental Water Samples Using a Vertically Ordered Mesoporous Silica Film and a Nanocarbon Composite

**DOI:** 10.3390/nano12203632

**Published:** 2022-10-16

**Authors:** Weiran Zheng, Ruobing Su, Guoguang Yu, Lin Liu, Fei Yan

**Affiliations:** 1 Institute of Agro-product Safety and Nutrition, Zhejiang Academy of Agricultural Sciences, Hangzhou 310021, China; 2 Key Laboratory of Surface & Interface Science of Polymer Materials of Zhejiang Province, Department of Chemistry, Zhejiang Sci-Tech University, Hangzhou 310018, China

**Keywords:** paraquat, electrochemically reduced graphene oxide, carbon nanotubes, vertically ordered mesoporous silica films, electrochemical detection

## Abstract

Herein, we demonstrate a sensitive and rapid electrochemical method for the detection of paraquat (PQ) using a glassy carbon electrode (GCE) modified with vertically ordered mesoporous silica films (VMSF) and a nanocarbon composite. The three-dimensional graphene-carbon nanotube (3DG-CNT) nanocarbon composite has a 3D network structure, a large electroactive area and oxygen-containing groups, promoting electron transfer between PQ and the underlying electrode and providing a suitable microenvironment for the stable growth of VMSF. This VMSF/3DG-CNT nanocomposite film could be prepared on the GCE’s surface by a two-step electrochemical method with good controllability and convenience. Owing to the synergistic effect of the electrocatalytic ability of 3DG-CNT and the electrostatically enriched capacity of VMSF, the proposed VMSF/3DG-CNT/GCE has superior analytical sensitivity compared with the bare GCE. Furthermore, VMSF has excellent anti-fouling ability that makes the fabricated sensor exhibit satisfactory performance for direct analysis of PQ in environmental water samples.

## 1. Introduction

Paraquat (1,1-dimethyl-4,4-bipyridine dichloride, PQ) is a kind of herbicide that has been widely used in a variety of applications, such as broad weed control and desiccants on crops [1,2]. Paraquat, also known as methyl viologen, is one of the active components of dipyridine compounds [3], which are easily reduced to stable free radicals and cause severe toxicity to humans and animals [4]. It has been proven that PQ may cause serious damage to the lungs, liver, kidney and heart, and also lead to neurodegenerative diseases such as Parkinson’s disease [5,6]. Overuse of PQ in agriculture will produce pollution in the environment (mainly the aquatic system). The maximum residue limit (MRL) for PQ in water is 3–200 nM [7]. Therefore, it is very important to realize the sensitive detection of PQ. Currently, many analytical methods have been devoted to the detection of PQ, such as chromatography [8], spectrophotometry [9], surface-enhanced Raman spectroscopy [10] and electrochemistry [11]. By contrast, electrochemical sensors have the advantages of high sensitivity, easy operation, rapidity and low cost, and have received considerable attention. Traiwatcharanon et al. [12] prepared lead oxide nanomaterials on SPE (PBO-NPs /SPE) by using the room-temperature spark method for electrochemical detection of PQ. Wachholz et al. [11] reported that a copper-based metal–organic framework/reduced graphene oxide-modified electrode (CuMOF/rGO/Au) could be used to detect PQ. Jiang et al. [13] deposited Au and Cu_2_O on the surface of an indium tin oxide (ITO) electrode for PQ detection. However, a complex electrode preparation process and sample pretreatments are often required, limiting the application of electrochemical sensors in real samples. Therefore, it is necessary to develop a simple, sensitive and anti-fouling electrochemical sensing interface for the detection of PQ.

Porous materials have been used as important functional building blocks for the development of various high-performance sensors and nanofluidic osmotic power generators [14,15,16], taking advantage of the high porosity and surface area [17,18], efficient enrichment ability [19,20] and rapid mass transfer [21], as well as their ease of hybridization [22,23,24]. In particular, vertically ordered mesoporous silica films (VMSF) consisting of ultrasmall and uniform pores (2~3 nm), perpendicular silica nanochannels and high porosity exhibit unique characteristics in terms of their good molecular accessibility, high permselectivity (e.g., size [25], charge [26] and lipophilicity [27]) and excellent anti-fouling capacity [28]. These have emerged as attractive electrode materials for the construction of electrochemical sensors [29,30,31]. Generally, Stöber solution growth approaches and electrochemically assisted self-assembly (EASA) are two simple and commonly used methods for the fabrication or further surface functionalization of VMSF onto the surface of electrodes [32,33,34]. In comparison with the former method, EASA is faster (around several seconds) and easier to operate, and the obtained VMSF has a better long-range order. Moreover, EASA can prepare VMSF on the surface of many conductive electrodes, such as gold, platinum, copper, indium tin oxide (ITO) and glassy carbon electrodes (GCE) [35,36,37]. Among these, GCE is one of most commonly used commercial electrodes and displays good electrochemically activity [38]. However, VMSF have poor adhesion on the untreated carbon-based electrodes. To overcome this issue, adhesive layers (e.g., organosilanes [39] and reduced graphene oxide [40]) and pretreatment process (e.g., electroactivation [41] and O_2_-plasma treatment [42]) are used. As our group reported, graphene with oxygen-containing groups, π-π structures and electrochemical activity could effectively improve the stability between VMSF and GCE, and simultaneously promote the electrode performance [40]. Furthermore, combining graphene with other nanomaterials (e.g., carbon nanotubes [43] and boron nitride [44]) could synergistically increase the specific surface area, conductivity and electroanalytical performance.

In this work, a three-dimensional nanocarbon composite composed of electrochemically reduced graphene oxide and carbon nanotubes (3DG-CNT) was used as a highly electroactive support for the stable growth of VMSF. These VMSF/3DG-CNT were prepared on the GCE’s surface by a two-step electrochemical method combining the electroactive ability of the inner 3DG-CNT layer and the electrostatic amplification effect of the outer VMSF layer, showing excellent electroanalytical performance towards PQ. The fabrication process of VMSF/3G-CNT/GCE sensors is simple, time-saving and controllable. Furthermore, owing to the good anti-fouling and anti-interference capacity of VMSF, the proposed VMSF/3DG-CNT/GCE sensor showed satisfactory results in environmental water samples, with improved stability and sensitivity.

## 2. Materials and Methods

### 2.1. Chemicals and Materials

All the chemicals and reagents were of analytical grade and were used as received without further purification. An aqueous solution of graphene oxide (GO) (1 mg/mL, 3~5 μm, evenness 99%, oxygen content 30~40%) was purchased from Hangzhou Gaoxi Tech. Multiwalled carbon nanotubes (CNT, OD < 8 nm, length~30 μm, 95%) were ordered from Chengdu Institute of Organic Chemistry, Chinese Academy of Sciences. Sodium perchlorate (NaClO_3_), sodium phosphate dibasic dodecahydrate (Na_2_HPO_4_·12H_2_O), sodium phosphate monobasic dihydrate (NaH_2_PO_4_·2H_2_O), paraquat (PQ), tetraethoxysilane (TEOS, 98%), potassium hydrogen phthalate (KHP), potassium ferricyanide (K_3_[Fe(CN)_6_]), cadmium nitrate (CdNO_3_·4H_2_O), catechol (CC), sodium dodecyl sulfate (SDS), starch and humic acid (HA) were bought from Aladdin. Hydrochloric acid (HCl) was obtained from Hangzhou Shuanglin Chemical reagent. Cetyltrimethylammonium bromide (CTAB), hydroquinone (HQ) and *p*-aminophenol (*p*-AP) were purchased from Macklin, and *p*-nitrophenol was obtained from Sarn Chemical Technology (Shanghai). Ethanol, sodium chloride (NaCl), calcium chloride (CaCl_2_), potassium chloride (KCl), ferric chloride (FeCl_2_) and magnesium chloride (MgCl_2_, 95%) were received from Hangzhou Gaojing Fine Chemical Industry. Bovine serum albumin (BSA) and hexaammonium ruthenium chloride (Ru(NH_3_)_6_Cl_3_, 98%) were ordered from Sigma Aldrich (Shanghai, China). All aqueous solutions were prepared with ultrapure water (18.2 MΩ cm). Pond water and soil were obtained from the campus of Zhejiang Sci-Tech University.

### 2.2. Measurements and Instrumentations

Scanning electron microscopy (SEM) measurements were performed on an SU8100 scanning electron microscope (Hitachi, Japan) with an accelerating voltage of 10 kV. X-photoelectron spectroscopy (XPS) data were obtained from a PHI5300 electron spectrometer (PE Ltd., Waltham, MA, USA) at 250 W, 14 kV and Mg Kα radiation. All electrochemical tests, including cyclic voltammetry (CV) and differential pulse voltammetry (DPV), were carried out on a PGSTAT302N Autolab electrochemical workstation (Metrohm, Herisau, Switzerland). The test adopted a conventional three-electrode system, including bare or modified GCE as the working electrode (the electrode’s size was 0.5 cm × 0.5 cm), a platinum sheet as the counter-electrode and an Ag/AgCl (saturated with KCl solution) electrode as the reference electrode. DPV test parameters were as follows: the step potential was 5 mV, the pulse amplitude was 25 mV, the pulse time was 0.05 s and the time interval was 0.2 s.

### 2.3. Preparation of 3DG-CNT/GCE and VMSF/3DG-CNT/GCE Electrodes

**Scheme 1:**
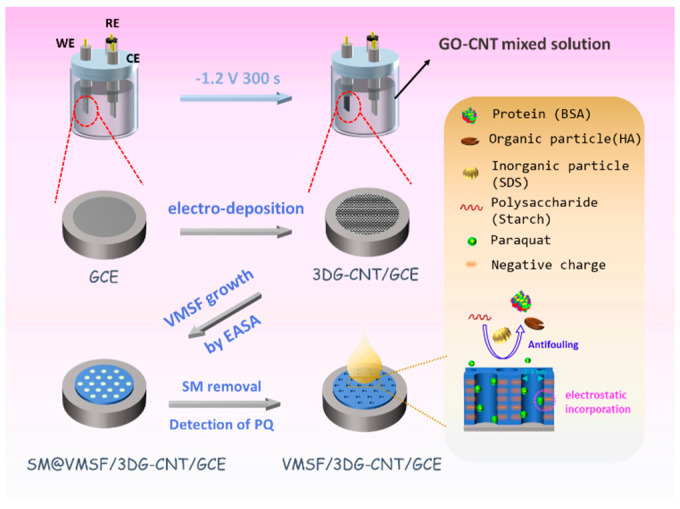
Illustration of the preparation of VMSF/3DG-CNT/GCE and the direct detection of PQ in complex environmental samples.

According to a previous literature report [45], 3DG-CNT were prepared on the GCE’s surface by using the electrochemical method (Scheme 1). GO-CNT dispersion was first achieved by mixing GO (3 mg mL^−1^) and CNT (0.3 mg mL^−1^) into 0.2 M NaClO_4_ with further sonication for 30 min. After being polished with 0.3 μm and 0.05 μm alumina powders and ultrasonically washed with ethanol and distilled water for 1 min each, a clean GCE was obtained. The GCE was soaked into the above GO-CNT dispersion and underwent a constant cathodic voltage of −1.2 V for 300 s with a platinum sheet electrode as the counter-electrode and an Ag/AgCl electrode (saturated with KCl) as the reference electrode. In this process, the GO was reduced to electrochemically reduced graphene oxide (ErGO), and a 3D nanocarbon composite consisting of graphene and CNT (termed 3DG-CNT) was deposited onto the GCE’s surface. Note that the CNTs, as electronic conducting wires or spacers, could not only facilitate the electrochemical reduction of GO but also greatly increased the active area of the electrode. The obtained 3DG-CNT/GCE had the advantages of both graphene and CNT.

VMSF were then grown on the 3DG-CNT/GCE using the EASA method [33]. Originating from the oxygen-containing groups of 3DG-CNT, VMSF formed a chemical bond with 3DG-CNT and displayed good stability on the GCE’s surface using 3DG-CNT as the adhesive nanolayer. Briefly, TEOS (2.833 g) and CTAB (1.585 g) were added to a mixed solution of ethanol (20 mL) and NaNO_3_ (20 mL, 0.1 M, pH = 2.6), followed by stirring for 2.5 h to prepare a silica precursor solution. Next, the platinum sheet, the Ag/AgCl electrode (saturated with KCl) and the 3DG-CNT/GCE were used as the counter-electrode, reference electrode and working electrode, respectively. These three electrodes were soaked in the precursor solution and the VMSF were prepared by applying a constant cathodic current density (–0.74 mA/cm^2^) to the 3DG-CNT/GCE for 10 s. Thus, a surfactant micelle (SM)-templated VMSF formed on the surface of 3DG-CNT/GCE after being washed with distilled water and aged at 80 °C for 10 h, termed SM@VMSF/3DG-CNT/GCE. The SM@VMSF/3DG-CNT/GCE was immersed in a 0.1 M HCL–ethanol solution and stirred for 5 min to remove SM, finally achieving the VMSF/3DG-CNT/GCE with open channels.

## 3. Results

### 3.1. Characterization of 3DG-CNT/GCE and VMSF/3DG-CNT/GCE

**Figure 1:**
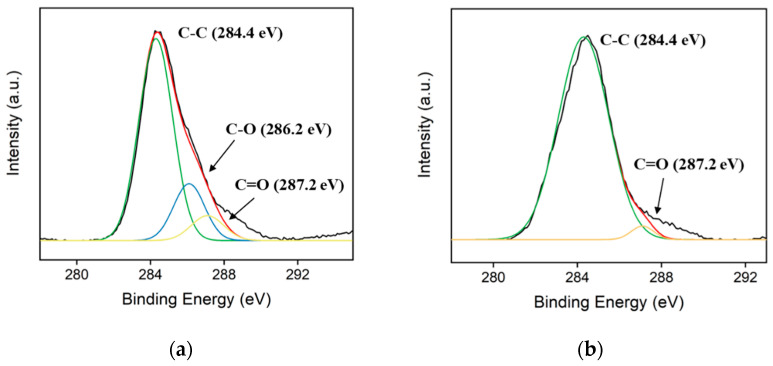

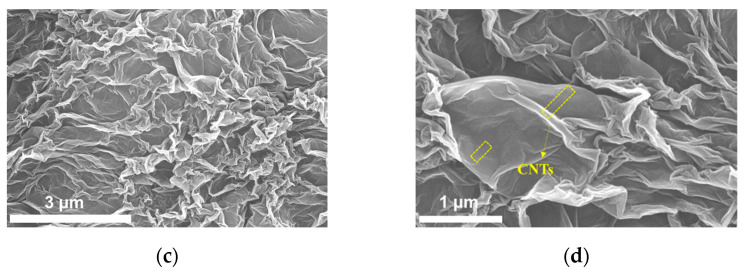
(**a**,**b**) The XPS C1s spectra of the GO (**a**) and ErGO (**b**). (**c**,**d**) SEM images of the 3DG-CNT network electrodeposited under a potential of −1.2 V for 300 s.

The process of reducing GO to ErGO was studied by XPS and the results are shown in Figure 1a,b. As demonstrated, the high-resolution C1s spectra of both GO and ErGO revealed three types of carbon bonds, including C–C/C=C (sp^2^ carbon, 284.4 eV), C–O (286.2 eV) and C=O (287.2 eV). When GO was electrochemically reduced to ErGO, the C-O peak could not be observed and the intensity of the C=O peak decreased, suggesting that the oxygen-containing functional groups of GO had been successfully reduced in the electrochemical process, and thus the graphene sheet had been reduced and removed. Figure 1c,d shows the SEM images of 3DG-CNT/GCE with different amplifications. As seen, there are many crumples perpendicularly aligned to the electrode’s surface in the 3DG-CNT, indicating the increased effective specific surface area of the electrode and the improved accessible mass transfer. In the high-resolution SEM image shown in Figure 1b, a small amount of CNT can be observed, showing the successful preparation of 3DG-CNT.

**Figure 2:**
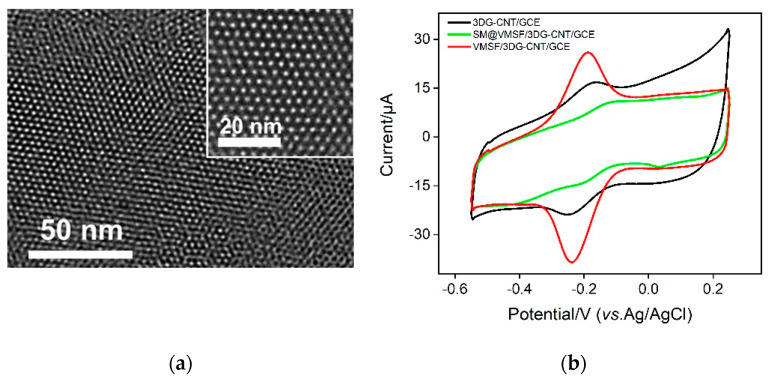
(**a**) Top-view TEM image of the VMSF. The inset is the corresponding magnified image. (**b**) CV curves obtained for different electrodes in a 0.05 M KHP solution containing 0.5 mM [Ru(NH_3_)_6_]^3+^.

To investigate the structure and morphology of the VMSF on the 3DG-CNT/GCE, TEM measurements were performed. As shown in Figure 2a, the VMSF had uniform pores with a long-range order and the diameter was 2~3 nm. The pores were hexagonally aligned and very regular over a large domain. Cyclic voltammetry (CV) is considered to be an effective method to evaluate the intactness and permselectivity of VMSF [46]. Figure 2b displays the CV curves of bare GCE, SM@VMSF/3DG-CNT/GCE and VMSF/3DG-CNT/GCE in a 0.05 M KHP solution containing 0.5 mM Ru(NH_3_)_6_^3+^. Due to the hinderance effect of the SM confined inside the silica nanochannels, only the charging current could be observed in the SM@VMSF/3DG-CNT/GCE. The VMSF/3DG-CNT/GCE with open channels produced a more obvious redox current peak compared with the bare GCE, showing the enrichment effect of cationic Ru(NH_3_)_6_^3+^. This arose from the negative surface of VMSF with deprotonated silanol groups, which confirmed the potential of VMSF for the detection of cationic species.

According to previous literature reports [47], the effective electroactive areas of bare GCE and VMSF/3DG-CNT/GCE could be calculated by using the Randles–Sevcik equation

*I_p_* = 0.4463*n*F*Ac*(*n*F*νD*/RT)^1/2^ (1)

where *I_p_* reflects the peak current, *n* is the number of transferred electrons, F is the Faraday constant (96,485 c·mol^−1^), *A* is the effective surface area, *c* is the concentration of K_3_[Fe(CN)_6_] (mM), *ν* is the scan rate (*ν*·s^–1^), *D* is the diffusion coefficient (6.67 × 10^−6^ cm^2^·s^–1^), R is the gas constant (8.314 J·mol^–1^K^–1^) and T is the Kelvin temperature (298 K).

**Figure 3:**
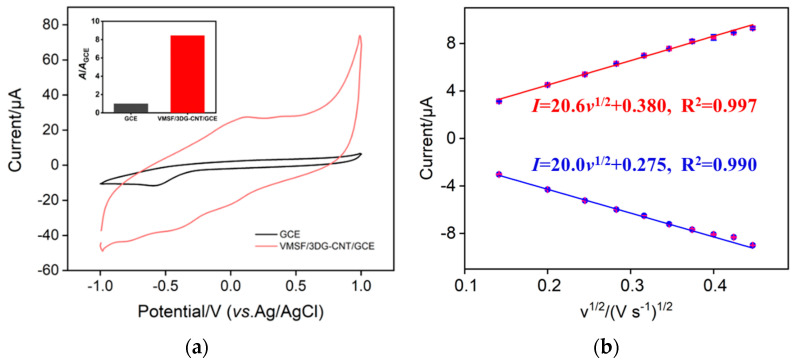
(**a**) CV curves of bare GCE (black) and VMSF/3DG-CNT/GCE (red) in PBS (0.1 M, pH = 6). The inset depicts the normalized electroactive surface area of each electrode. (**b**) Relationship between the peak currents and the square root of the scan rate derived from the CV curves of bare GCE in 0.05 M KHP containing 0.5 mM K_3_[Fe(CN)_6_].

Figure 3a shows the CV curves of the bare GCE and the VMSF/3DG-CNT/GCE in 0.1 M PBS (pH = 6). The pair of redox peaks around 0 V is due to the electrochemical reaction between the quinone and hydroxyquinone moieties in the 3DG-CNT. The cathodic current peak displayed at around –0.5 V is ascribed to the reduction of oxygen (Figure S1). As a comparison, the surface area of the VMSF/3DG-CNT/GCE is about 8.5 times larger than that of bare GCE, which is due to the formation of the 3D network structure of the 3DG-CNT nanocomposite. The effective surface area of bare GCE was calculated to be 0.0935 cm^2^, according to the data shown in Figure 3b and Equation (1). Thus, the effective surface area of the VMSF/3DG-CNT/GCE was about 0.790 cm^2^.

### 3.2. Electrochemical Behavior of PQ on the VMSF/3DG-CNT/GCE

**Figure 4:**
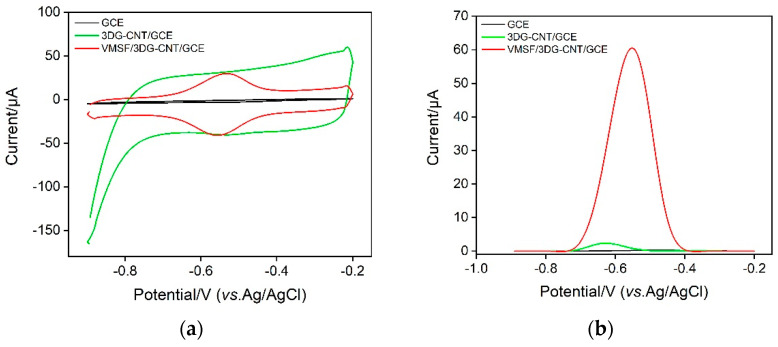
(**a**) CV and (**b**) DPV curves obtained with different electrodes in 0.1 M PBS (pH = 6) containing a 10 μM PQ solution.

**Scheme 2:**
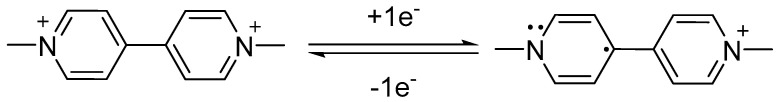
Schematic diagram of PQ’s reaction process.

In order to verify the feasibility and performance of the VMSF/3DG-CNT/GCE for the determination of PQ, we compared the CV and DPV responses of the bare GCE, 3DG-CNT/GCE and VMSF/3DG-CNT/GCE electrodes to PQ, and the results are shown in Figure 4. As displayed in Figure 4a, PQ showed a weak or no electrochemical response by the bare GCE and 3DG-CNT/GCE, but showed a pair of obvious reversible redox peaks with the VMSF/3DG-CNT/GCE, which involve a single-electron transfer reactions (Scheme 2) [48]. Moreover, the magnitude of the anodic peak current of PQ on the VMSF/3DG-CNT/GCE was around 15-fold greater than that of 3DG-CNT/GCE and was much higher than that of the bare GCE (Figure 4b). This excellent performance of the as-prepared VMSF/3DG-CNT/GCE is attributed to the electrocatalytic ability of the 3DG-CNT and the electrostatically enriched capacity of VMSF.

### 3.3. Electrochemical Detection of PQ Using a VMSF/3DG-CNT/GCE Sensor

**Figure 5:**
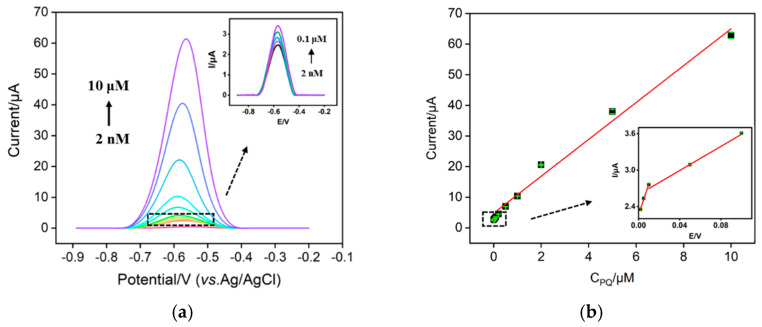
(**a**) DPV curves of the VMSF/3DG-CNT/GCE obtained in PBS (0.1 M, pH = 6) containing different concentrations of PQ. The inset is the magnified view of the DPV curves in the low concentration region. (**b**) The calibration curve for PQ. The inset in (**b**) shows the calibration lines in the low concentration range. The error bars represent the standard deviation (SD) of three measurements.

In order to achieve a highly sensitive performance for the detection and preparation PQ, the detection conditions, including the electrodeposition time of the 3DG-CNT, the growth time of the VMSF, the pH and the concentration of the supporting electrolyte and preconcentration time, were optimized. These results are shown in Figures S2.1–2.5 in the Supplementary Materials. As seen, a 3DG-CNT electrodeposition time of 300 s, a VMSF growth time of 10 s, 0.1 M PBS (pH = 6) and mechanical stirring for 180 s were the optimal experimental conditions. Next, the VMSF/3DG-CNT/GCE sensor was used to electrochemically detect PQ at various concentrations under optimized conditions. As revealed in Figure 5, when the PQ concentration varied from 2 nM to 10 μM, the anodic peak current (*I*) gradually increased and had a good linear relationship with the PQ’s concentration (*C*_PQ_), yielding two linear ranges of 2 nM–10 nM and 10 nM–10 μM. The linear regressive equations in the low and high concentration ranges are *I* = 45.83 *C*_PQ_ + 2.317 (*R*^2^ = 0.987) and *I* = 8.129 *C*_PQ_ + 2.675 (*R*^2^ = 0.997), respectively. The limit of detection (LOD) was 1.17 nM when the signal-to-noise ratio was 3 (S/N = 3). Table 1 lists the performance of different electrochemical sensors for the detection of PQ. By comparison, our sensor has a rather lower LOD and a wider linear range, showing good analytical performance.

### 3.4. The Anti-Interference and Repeatability of the VMSF/3DG-CNT/GCE Sensor

The anti-interference ability of the VMSF/3DG-CNT/GCE electrode was investigated. Common interfering substances in the environment, such as metal ions (Na^+^, K^+^, Cd^2+^, Mg^2+^ and Cu^2+^), and other possible small electroactive molecules and environmental pollutants (hydroquinone (HQ), catechol (CC), *p*-aminophenol (*p*-AP) and p-nitrophenol (*p*-NP)) were added to the detection solution containing 1 μM PQ. As shown in Figure S3.1A, when 100 μM of metal ions or 10 μM of electroactive molecules was present in the detected solution, almost no effect on the detection of PQ was observed. It was found that Cu^2+^, HQ, CC, *p*-AP and *p*-NP had no anodic current peaks in the detected potential window of paraquat and the voltammetric peak of Cd^2+^ was around −0.77 V, which is far from that of paraquat (−0.58 V), indicating the good anti-interference ability of VMSF/3DG-CNT/GCE. In addition, the repeatability of the VMSF/3DG-CNT/GCE electrode was evaluated. VMSF/3DG-CNT/GCE was used to repeatedly detect 1 μM PQ, and the anodic peak currents obtained from five measurements were almost same (Figure S3.2), showing the excellent repeatability of the proposed sensor. After 5 days of storage, our VMSF/3DG-CNT/GCE sensor displayed good stability (Figure S3.3).

### 3.5. Real Sample Analysis

**Figure 6:**
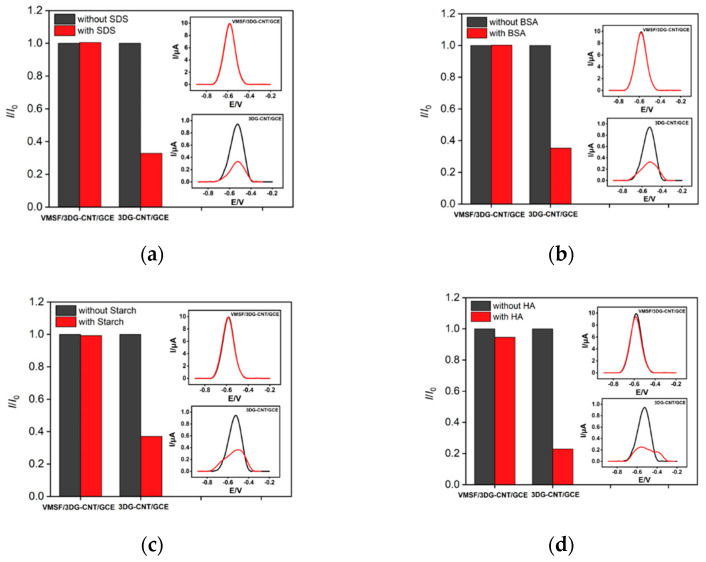
Normalized oxidation peak current ratio of the VMSF/3DG-CNT/GCE and 3DG-CNT/GCE towards PQ (1 μM). *I* and *I*_0_ represent the currents obtained in the present and absence of 20 μg/mL of SDS (**a**), BSA (**b**), starch (**c**) or HA (**d**) in 0.1 M PBS (pH = 6). The insets are the corresponding DPV curves obtained for the 3DG-CNT/GCE and VMSF/3DG-CNT/GCE in the absence and presence of the fouling species.

The anti-fouling ability of the VMSF/3DG-CNT/GCE is very important for the analysis of real samples. Surfactants (sodium dodecyl sulfate, SDS), protein (bovine serum albumin, BSA), polysaccharide (starch) and macromolecular substances (humic acid, HA) commonly exist in complex samples and may produce severe surface fouling of an electrode. These were used to verify the anti-fouling ability of the VMSF/3DG-CNT/GCE. We compared the anodic peak currents of PQ on the VMSF/3DG-CNT/GCE and 3DG-CNT/GCE with (*I*) and without (*I*_0_) fouling substances. As exhibited in Figure 6, the presence of fouling substances could generate surface fouling of the 3DG-CNT/GCE and passivate the sensor, leading to remarkably decreased peak currents (only 20~40% of the initial values) and much wider current peaks. However, 95% of the initial values still remained for the VMSF/3DG-CNT/GCE, indicating the excellent anti-fouling capacity of the VMSF/3DG-CNT/GCE and the great potential for the detection of PQ in complex samples.

Environmental water samples including pond water and a soil leaching solution were used to examine the applicability of the proposed VMSF/3DG-CNT/GCE. Pond water was diluted 10-fold with 0.1 M PBS (pH = 6). Moreover, 0.05 g of soil was added to 50 mL of PBS (pH = 6) to obtain a soil dispersion and the supernatant was directly used as a soil leaching solution for analysis of a real sample. The detection samples were first spiked with several known concentrations of PQ and examined by our VMSF/3DG-CNT/GCE sensor. By comparing the ratio of the concentration detected and the known concentration (recovery), we found that the recoveries achieved are 94.5–109% and the relative standard deviations (RSD) were within 2.3% (Table 2), suggesting the good accuracy and reliability of the VMSF/3DG-CNT/GCE for the determination of PQ in environmental water samples.

## 4. Conclusions

In summary, VMSF/3DG-CNT were prepared on the GCE’s surface by using a simple and controllable electrochemical method. The obtained VMSF/3DG-CNT/GCE sensor had several advantages. Firstly, the 3DG-CNT nanocarbon composite with a 3D network structure, good conductivity and oxygen-containing functional groups not only displayed a high electroactive area and excellent electrocatalytic ability, but also provided a suitable microenvironment for the stable growth of VMSF. Second, the electrocatalytic capacity of the inner 3DG-CNT layer and the electrostatic enrichment ability of VMSF were combined, showing superior analytical performance regarding PQ in terms of a low LOD and a wide linear range. Thirdly, given the inherent anti-fouling ability of VMSF, the VMSF/3DG-CNT/GCE was successfully applied for the detection of PQ in environmental water samples. Lastly, the fabrication of VMSF/3DG-CNT/GCE sensors is easy, economic and controllable, and could be extended to detect other varieties of analytes by simple functionalization of the 3DG-CNT or VMSF.

## Figures and Tables

**Table 1:** Comparison of the determination of PQ among different methods.

Electrode Materials	Method	Detection Range (μM)	Sensitivity (μA μM^–1^ cm^–2^)	LOD (nM)	Refs.
AuNP-MWCNT/GCE	SWV	1–2	–	32	[49]
VMSF/GCE	SWV	0.01–0.05	42	12	[48]
BW-CB/GCE	DPV	0.5–7.5	0.487	–	[50]
BN/MoS_2_/Au NPs/GCE	DPV	0.1–100	4.44 × 10^−5^	74	[3]
SPCE-CNT/Nafion	DPV	0.54–4.3	–	170	[1]
PPY-g-NGE/GCE	DPV	0.05–2	–	41	[51]
Micro-Cu_2_O/PVP-GNs/GC-RDE	DPV	1–200	3.00 × 10^–5^	260	[52]
VMSF/3DG-CNT/GCE	DPV	0.002–0.01 0.01–10	0.0578 0.0103	1.17	This work

AuNP, Au nanoparticle; MWCNT, multiwalled carbon nanotubes; SWV, square wave voltammetry; BW, beeswax; CB, carbon black; DPV, differential pulse voltammetry; BN, hexagonal boron nitride; MoS_2_, molybdenum disulfide; SPCE, screen-printed carbon electrode; SI-DPV, sequential injection-differential pulse voltametric; PPY-g, polypyrrole-grafted; NGE, nitrogen-doped graphene; Micro-Cu_2_O, cuprous oxide sub-microparticles; PVP, polyvinyl pyrrolidone; GNs, graphene nanosheets; GC-RED, glassy carbon-rotating disk electrode.

**Table 2:** Determination of PQ in pond water and soil leaching solution samples.

Sample	Spiked (μM)	Found (μM)	RSD (%, *n* = 3)	Recovery (%)
Pond water	0.0500	0.0499	1.8	99.8
	0.100	0.109	1.8	109
	0.200	0.199	2.3	99.5
Soil leaching solution	0.100	0.100	2.3	100
	0.200	0.189	3.3	94.5
	0.300	0.313	3.3	104

## Data Availability

The data presented in this study are available on request from the corresponding author.

## Supplementary Materials

The following supporting information can be downloaded at https://www.mdpi.com/article/10.3390/nano12203632/s1. Figure S1: CV curves of bare GCE and VMSF/3DG-CNT/GCE in deoxygenated PBS; Figure S2.1: Optimization of electrodeposition time of 3DG-CNT; Figure S2.2: Optimization of growth time of VMSF; Figure S2.3: Optimization of pH of supporting electrolyte. Figure S2.4: Optimization of concentration of supporting electrolyte; Figure S2.5: Optimization of preconcentration time. Figure S3.1: Anti-interference ability of VMSF/3DG-CNT/GCE; Figure S3.2: Repeatability of VMSF/3DG-CNT/GCE; Figure S3.3: Stability of VMSF/3DG-CNT/GCE.
