# Supplementary material for: Highly Sensitive Electrochemical Detection of Paraquat in Environmental Water Samples Using a Vertically Ordered Mesoporous Silica Film and a Nanocarbon Composite
Source: Nanomaterials (Basel). 2022 Oct 16;12(20):3632. doi: 10.3390/nano12203632 (PMC9610481; doi:10.3390/nano12203632)
Supplement: Supplementary file 1 [file nanomaterials-12-03632-s001.zip › nanomaterials-1936351-supplementary.pdf]

nanomatericals

Supplementary Materials

# Highly Sensitive Electrochemical Detection of Paraquat in Environmental Water Samples Using a Vertically Ordered Mesoporous Silica Film and a Nanocarbon Composite

**Weiran Zheng [1,*], Ruobing Su [2], Guoguang Yu [1], Lin Liu [1] and Fei Yan [2,*]**

[1] Institute of Agro-product Safety and Nutrition, Zhejiang Academy of Agricultural Sciences, Hangzhou 310021, China
[2] Key Laboratory of Surface & Interface Science of Polymer Materials of Zhejiang Province, Department of Chemistry, Zhejiang Sci-Tech University, Hangzhou 310018, China
* Correspondence: rancki@163.com (W.Z.); yanfei@zstu.edu.cn (F.Y.)

### S1 CV curves of bare GCE and VMSF/3DG-CNT/GCE in deoxygenated PBS

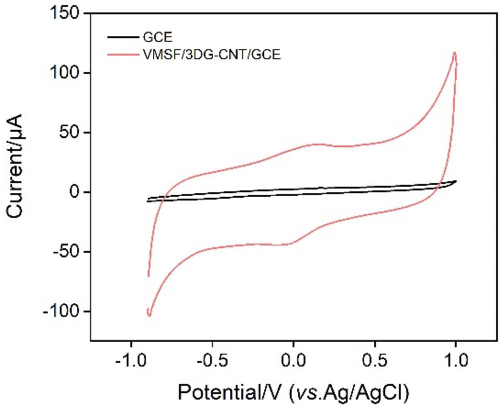


**Figure S1.** CV curves of bare GCE (black) and VMSF/3DG-CNT/GCE (red) in deoxygenated PBS (0.1 M, pH = 6).

## S2 Optimization of experimental conditions

*S2.1 Electrodeposition time of 3DG-CNT*

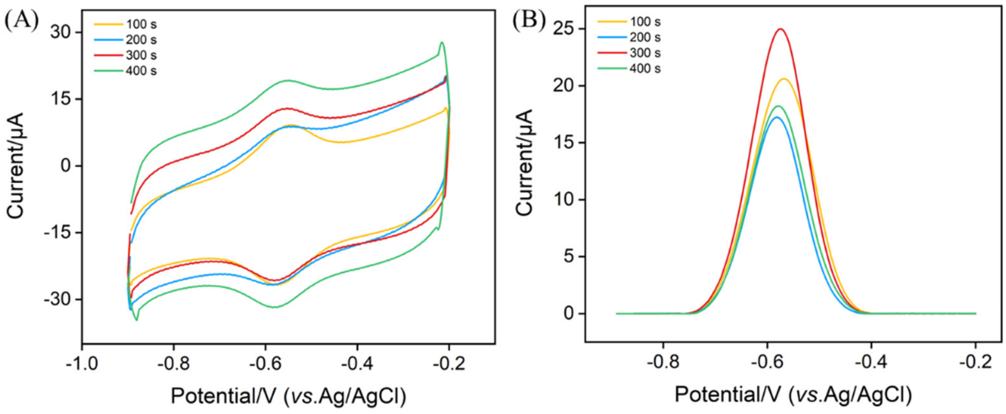


**Figure S2.1** (**A**) CVs and (**B**) DPVs obtained at the 3DG-CNT/GCE electrodeposited under the potential of −1.2 V for various times in 0.1 M PBS (pH = 6) containing 3.5 μM PQ.

*S2.2 Growth time of VMSF*

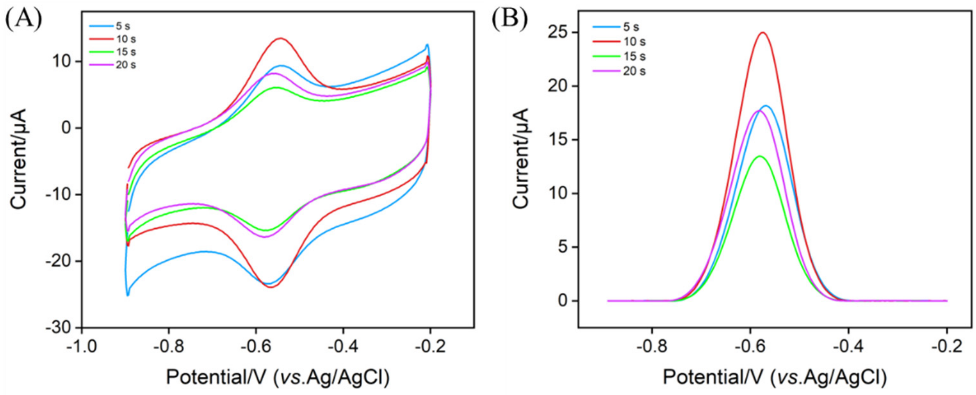


**Figure S2.2** (**A**) CVs and (**B**) DPVs obtained at the VMSF/3DG-CNT/GCE under different growth times of VMSF in 0.1 M PBS (pH = 6) containing 3.5 μM PQ.

*S2.3 pH of supporting electrolyte*

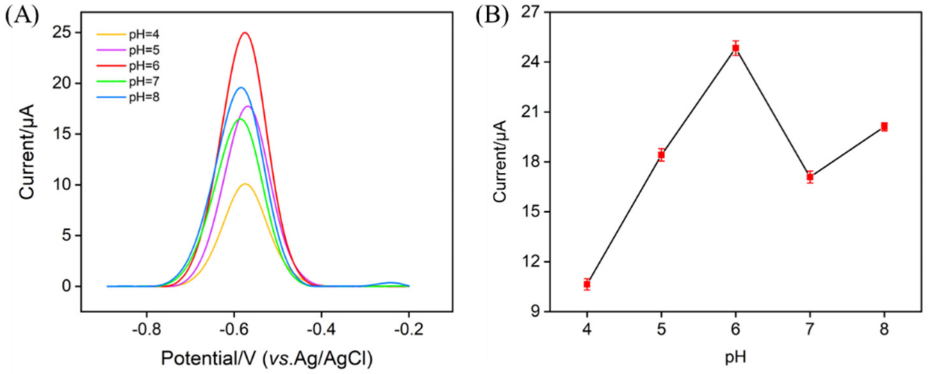


**Figure S2.3** (**A**) DPV curves obtained at the VMSF/3DG-CNT/GCE in 0.1 M PBS containing 3.5 μM PQ at various pH values. (**B**) Effect of the pH value of PBS on the detection of PQ. The error bars represent the SD of three measurements.

*S2.4 Concentration of supporting electrolyte*

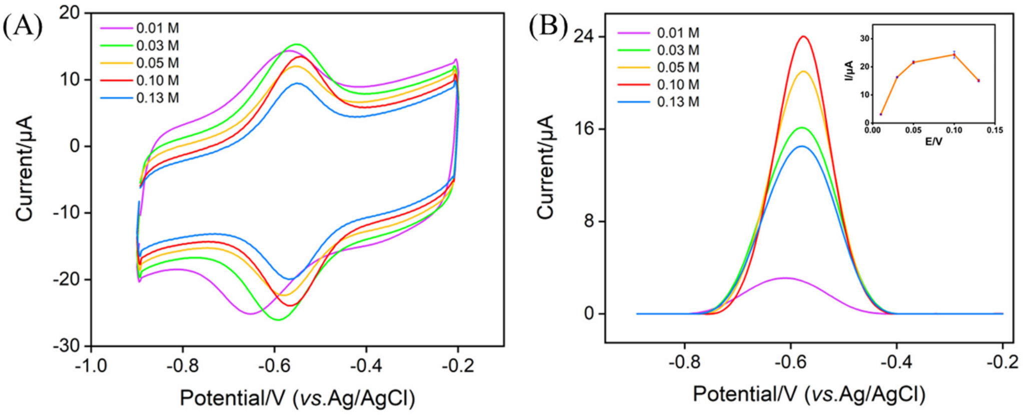


**Figure S2.4** (**A**) CVs and (**B**) DPVs obtained at the VMSF/3DG-CNT/GCE in response to 3.5 μM PQ at various concentrations of PBS (pH = 6). Inset is the concentration of PBS on the detection of PQ. The error bars represent the SD of three measurements.

*S2.5 Preconcentration time*

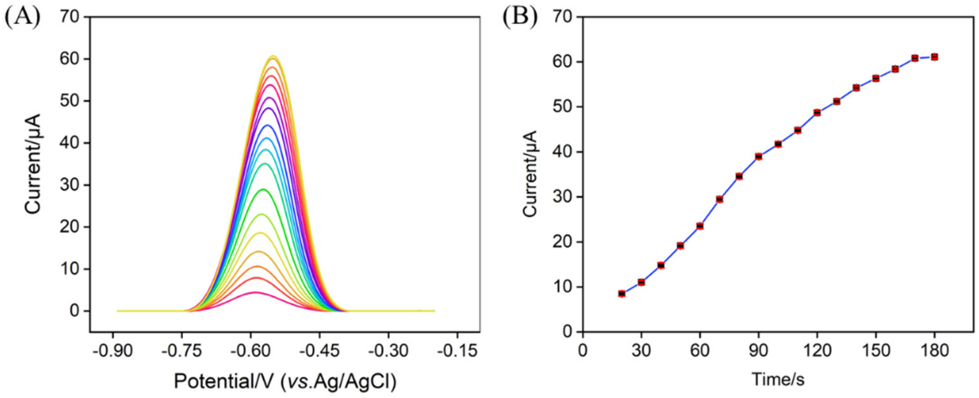


**Figure S2.5** (**A**) DPVs obtained at the VMSF/3DG-CNT/GCE in 0.1 M PBS (pH = 6) containing 10 μM PQ at various mechanical stirring time. (**B**) The dependence of anodic peak current on the preconcentration time.

## S3 Anti-interference ability, repeatability and stability of VMSF/3DG-CNT/GCE

### *S3.1 Anti-interference ability of VMSF/3DG-CNT/GCE*

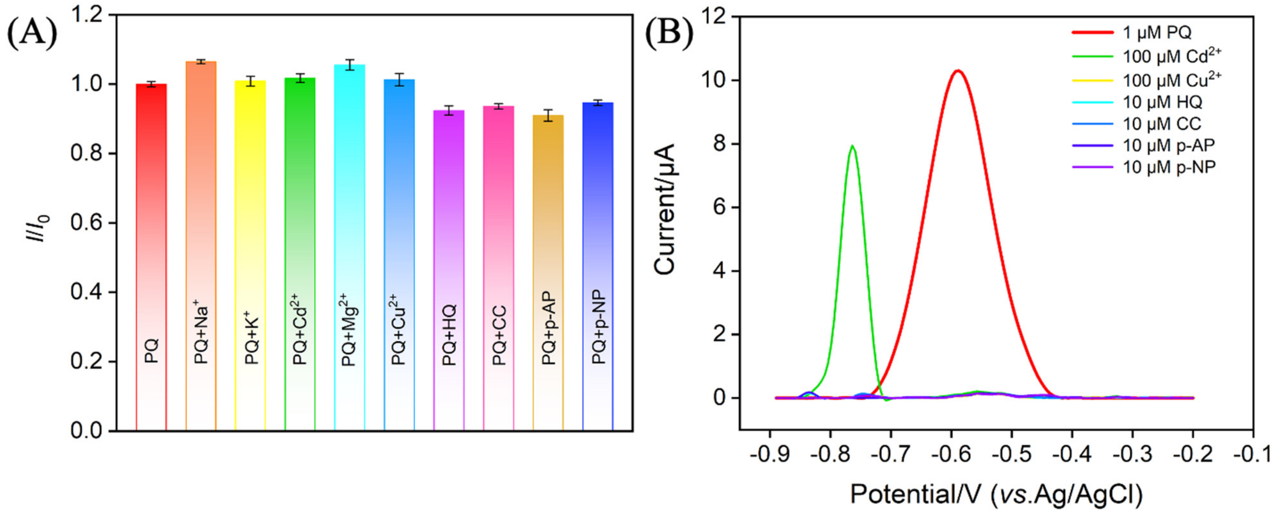


**Figure S3.1** (**A**) The current ratio ($I/I_0$) obtained from VMSF/3DG-CNT/GCE for detection of PQ (1 μM) in the absence ($I_0$) and presence ($I$) of 100-fold ($Na^+$, $K^+$, $Cd^{2+}$, $Mg^{2+}$, and $Cu^{2+}$) or 10-fold of HQ, CC, *p*-AP and *p*-NP. The error bars represent the SD of three measurements. (**B**) DPV curves of the VMSF/3DG-CNT/GCE for detection of PQ, $Cd^{2+}$, $Cu^{2+}$, HQ, CC, *p*-AP or *p*-NP.

### *S3.2 Repeatability of VMSF/3DG-CNT/GCE*

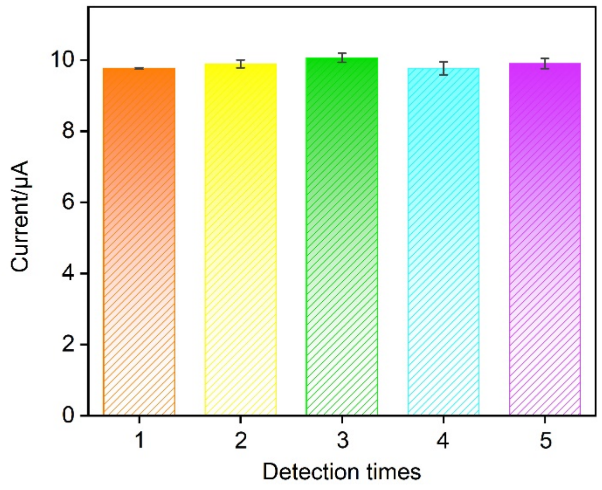


**Figure S3.2** Repeatability of VMSF/3DG-CNT/GCE in the presence of 1 μM PQ.

*S3.3 Stability of VMSF/3DG-CNT/GCE*

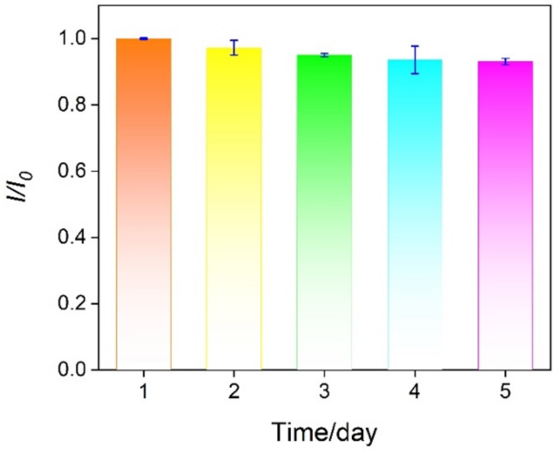


**Figure S3.3** Stability of the VMSF/3DG-CNT/GCE. $I$ and $I_0$ denote the anodic peak current measured at various days and initial measured value, respectively.
